# Exploring the Impact of Climate Variability on Malaria Transmission Using a Dynamic Mosquito-Human Malaria Model

**DOI:** 10.2174/1874279301810010088

**Published:** 2018-07-24

**Authors:** Gbenga J. Abiodun, Peter J. Witbooi, Kazeem O. Okosun, Rajendra Maharaj

**Affiliations:** 1 Research Unit, Foundation for Professional Development, Pretoria 0184, Republic of South Africa; 2 Department of Mathematics and Applied Mathematics, University of the Western Cape, Private Bag X17, Bellville7535, Republic of South Africa; 3 Department of Mathematics, Vaal University of Technology, X021, Vanderbijlpark, 1900, Republic of South Africa; 4 Office of Malaria Research, South African Medical Research Council, Durban, Republic of South Africa

**Keywords:** Climate variability, Mathematical model, *Anopheles arabiensis*, Malaria incidence, South Africa, Malaria dynamics

## Abstract

**Introduction:**

The reasons for malaria resurgence mostly in Africa are yet to be well understood. Although the causes are often linked to regional climate change, it is important to understand the impact of climate variability on the dynamics of the disease. However, this is almost impossible without adequate long-term malaria data over the study areas.

**Methods:**

In this study, we develop a climate-based mosquito-human malaria model to study malaria dynamics in the human population over KwaZulu-Natal, one of the epidemic provinces in South Africa, from 1970-2005. We compare the model output with available observed monthly malaria cases over the province from September 1999 to December 2003. We further use the model outputs to explore the relationship between the climate variables (rainfall and temperature) and malaria incidence over the province using principal component analysis, wavelet power spectrum and wavelet coherence analysis. The model produces a reasonable fit with the observed data and in particular, it captures all the spikes in malaria prevalence.

**Results:**

Our results highlight the importance of climate factors on malaria transmission and show the seasonality of malaria epidemics over the province. Results from the principal component analyses further suggest that, there are two principal factors associated with climates variables and the model outputs. One of the factors indicate high loadings on Susceptible, Exposed and Infected human, while the other is more correlated with Susceptible and Recovered humans. However, both factors reveal the inverse correlation between Susceptible-Infected and Susceptible-Recovered humans respectively. Through the spectrum analysis, we notice a strong annual cycle of malaria incidence over the province and ascertain a dominant of one year periodicity. Consequently, our findings indicate that an average of 0 to 120-day lag is generally noted over the study period, but the 120-day lag is more associated with temperature than rainfall. This is consistence with other results obtained from our analyses that malaria transmission is more tightly coupled with temperature than with rainfall in KwaZulu-Natal province.

## 1. INTRODUCTION

Malaria continues to be a serious concern as it causes almost one million deaths globally every year [1], It is also estimated that approximately half of the world’s population is at risk of contracting malaria [1]. The disease is closely associated with Africa, which is classified as a region carrying the largest share of the global malaria burden [1]. However, malaria is sensitive to climatic conditions and the occurrence is strongly influenced by climate variability [2]. For this reason, the recent concerns about global warming have triggered several studies on the impact of climate (variability and change) on inter-annual patterns of the disease [3–6].

Previous studies have examined year-to-year variation of seasonal epidemics over the African highlands [7], For example, Hay *et al* [8] confirmed the existence of cycle’s periodicity more than one year. Using the time-series modelling approach, Zhou *et al* [9] recently ascertained that rainfall and temperature play a significant role in the inter-annual variability of malaria across multiple East African highlands. Their results in contrast to [8] suggested that malaria epidemics in the highlands are initiated by climate variability. More recently, Pascual *et al* [7] combined both a time-series epidemiological model and a statistical approach to analyse monthly cases of malaria from 1970 to 2003 over a highland in Western Kenya. The findings from their study reveal the existence of multiyear cycles of malaria incidence over the study period. Their findings also demonstrate the impact of rainfall over malaria resurgence in 1990. It is concluded in line with the study of [9] that climate variables play significant roles at different temporal scales and should be considered when building predictive malaria models.

However, assessing the impact of climate variability on malaria transmission over a region is difficult without having a long-term data series of malaria cases of the region [9], For this reason, several studies have considered using dynamical malaria models to generate reported cases of epidemic regions over a long period. For instance, in the study of Laneri *et al* [10], dynamical models are used to understudy the impact of climate variability and immunity on malaria transmission in Senegal over two decades. Their results highlight the chances of predicting malaria incidence on endemic regions after investigating the interaction between climate and immunity. Following the same trend, Roy *et al* [11] examined the impact of climate variability on malaria and predicted epidemic regions over various districts in India with the help of dynamical models. It is concluded in their study that climate variables and process-based models are useful under non-stationary conditions. Okuneye and Gumel [12] designed a new non-autonomous model to assess the impact of variability in temperature and rainfall on the transmission dynamics of malaria in KwaZulu-Natal province of South Africa. Similarly, in the recent study of Abiodun *et al* [13], a climate-based mosquito model was presented to explore the impact of temperature and rainfall on mosquito population dynamics over Dondotha village in KwaZulu-Natal province, South Africa. The model demonstrates and quantifies the influence of temperature and rainfall on the abundance of *Anopheles arabiensis* over time and presents the strong seasonal variability over the region.

The current study aims to further develop the mosquito model presented in [13] to investigate the impact of climate variability on malaria transmission over KwaZulu-Natal province during the period 1970-2005. The newly developed mosquito-human malaria model will be used to analyse the temporal dynamics of the diseases over the province.

## 2. MATERIALS AND METHODS

### 2.1. Study Area

**Fig. (1).**
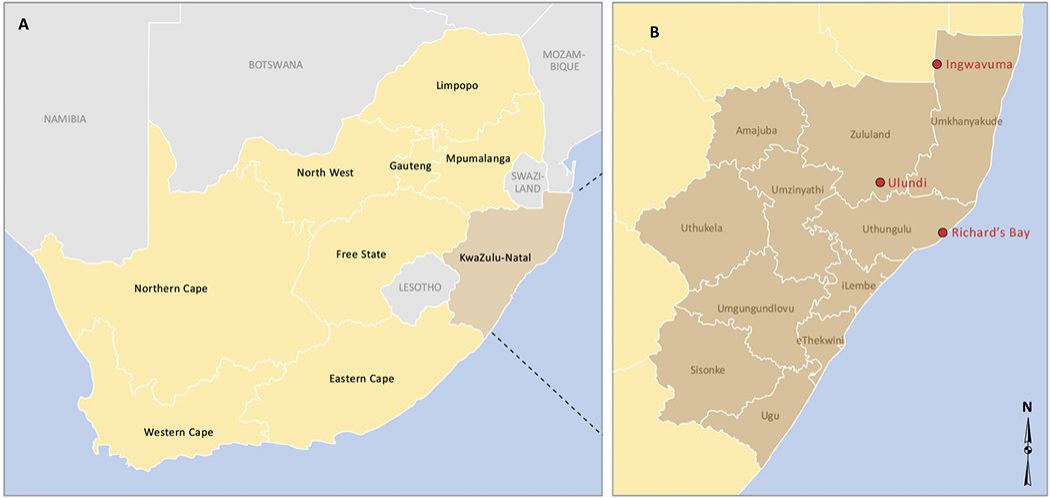
The map of KwaZulu-Natal province, South Africa. Source: GIS unit of the Medical Research Council of South Africa.

KwaZulu-Natal is a province located at the northeast part of South Africa. It is surrounded by three countries (Mozambique, Swaziland and Lesotho) and provinces (Mpumalanga, Free State and Eastern Cape), as in Fig. (1A and B). The province with almost 600,000 individuals living in malaria-risk areas, witnessed the highest malaria prevalence in 2001 in South Africa [14].

### 2.2. Climate and Malaria Data

**Fig. (2).**
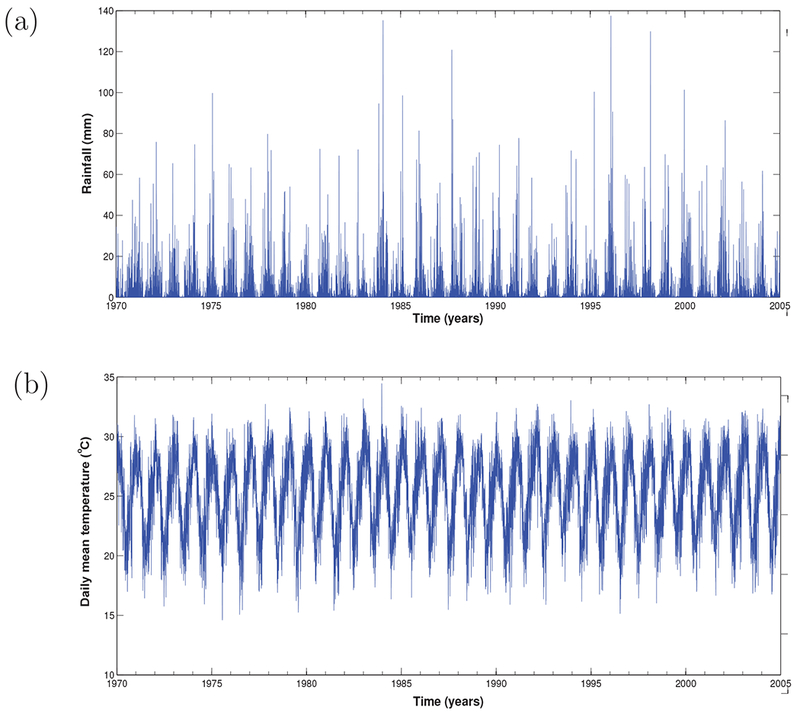
Time series of (a) daily mean temperature, and (b) rainfall of KwaZulu-Natal province from 1970 - 2005.

Two different datasets were considered for the purpose of this study. To estimate the climate data of KwaZulu-Natal province, we averaged the climate data of three towns within malaria risk areas in the province. The towns namely; Ingwavuma (27.1322°S, 31.9942°E), Richards Bay (28.7807°S, 32.0383°E) and Ulundi (28.2997°S, 31.4342°E) are selected from Umkhanyakude, Uthungulu, and Zululand districts respectively (Fig. 1B). The Observational-Reanalysis hybrid datasets of each town is obtained from the Princeton University Global Meteorological Forcing Datasets ((PUGMFD), see [15] for details), and consist of the daily precipitation, minimum and maximum temperatures from 1970–2005. The averaged climate data of the three towns for the daily mean temperature and rainfall over the study period is shown in Fig. (2A and B) respectively. The monthly provincial malaria cases data of KwaZulu-Natal between September 1999 and 2003 were obtained from the South African Department of Health.

### 2.3. Mosquito-Malaria Model Formulation

**Fig. (3).**
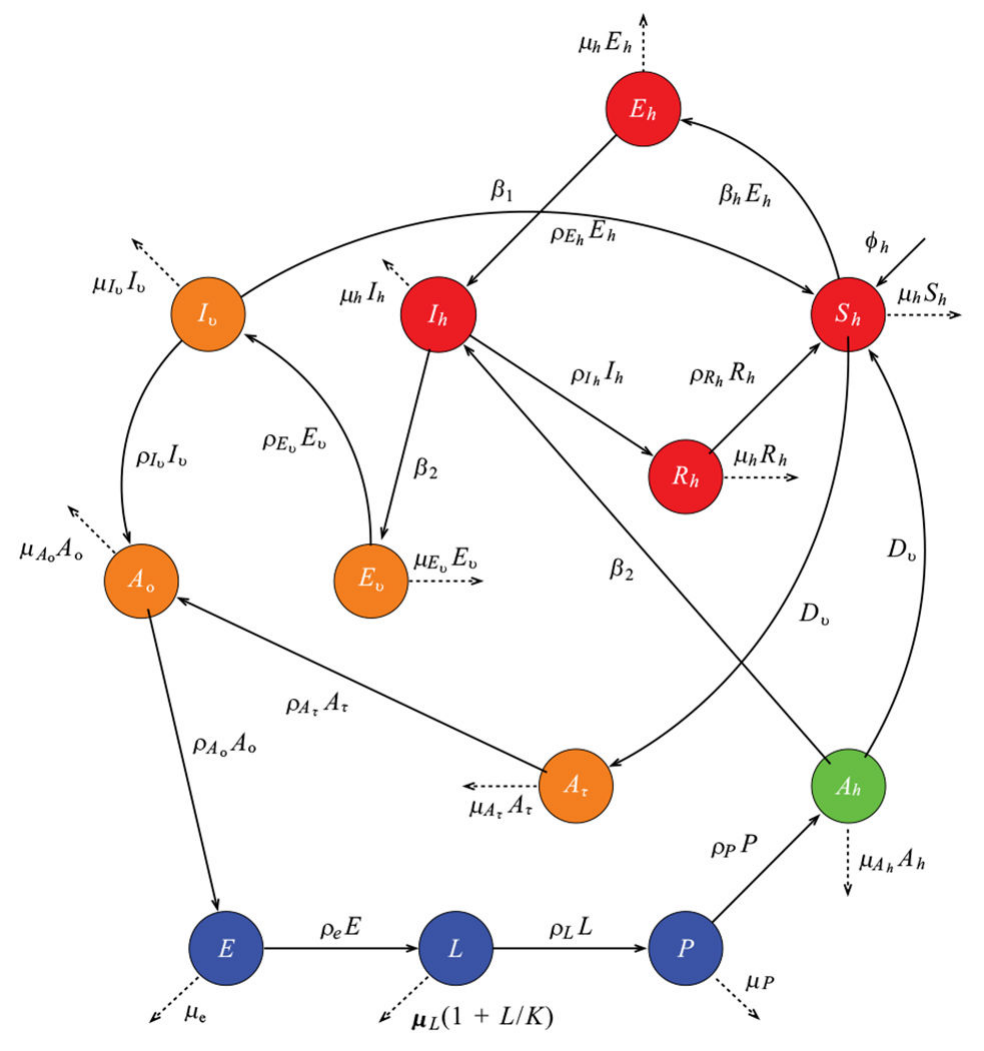
Flow diagram of the mosquito-human malaria model.

In this study, we develop a climate-based mosquito-human malaria model to examine malaria transmission over KwaZulu-Natal province. We incorporate into the mosquito model of [13], as described in [16] and in Table 1, where *N_h_* is the total human population, consisting of Susceptible *S_h_* Exposed Infected *I_h_* and Recovered *R_h_* individuals. The total population of mosquito, denoted by *N_h_* consist of Eggs (*E*), Larvae (*L*), Pupae *(P),* Susceptible adult searching for host (*A_h_*). Adult at resting state (*A_r_*). Adult searching for oviposition site(*A_o_*), Exposed adult (*E_v_*) and Infected adult mosquitoes (*I_v_*). We assume that a portion of *A_h_* will feed on *S_h_* while some feed directly on infected human *I_h_* Those that fed on susceptible human will proceed to adult resting (*A_r_*) to digest their meal, and later proceed to adult seeking oviposition site (*A_o_*) to lay their eggs, while those that fed on infected human proceed to exposed mosquito (*E_v_*)to digest their meal, and later proceed to Ao after leaving the infected stage (*I_v_*). The dynamics of the mosquito-human malaria model are described by the following system of differential equations (1) with the flow diagram illustrated in Fig. (3). The mosquito climate-dependent functions considered for this model are generated from the laboratory experiments of [33], For details on this, and on the mathematical analyses of the model in the present study, we refer readers to [13, 16], Parameters are estimated and adopted from other studies as shown in Table 1.

Since mosquitoes and malaria parasites respond to weather conditions in days [2], the impact of climate variables on malaria transmission will be underestimated when using a monthly dataset. For this reason, we run our model with daily climate data to simulate the daily human population dynamics over the study region. Considering the parameters in Table 1, we chose the following initial conditions for the model simulations; *S_h_* = 1000000, *E_h_* = 600, *I_h_* = 250 , *R_h_* = 120, *E* = 10000000, *L* = 80000000, *P* = 6000000, *A_h_* = 5000000, *A_r_* = 4000000, *A_o_* = 10000, *E_v_* = 8000, *I_v_* = 5000. Although the model output is obtained in daily basis, in order to ascertain the validity of the simulated data, we calculate the monthly number of infected humans over the province between September 1999 and December 2003 and compare our results with the observed monthly malaria cases over the province.

(1) $$\begin{array}{cc} \frac{{\mathrm{dS}}_{h}}{\mathrm{dt}}= & \Phi_{h}+\rho_{R_{h}}R_{h}-(\beta_{h}+\mu_{h})S_{h}\\ \frac{{\mathrm{dE}}_{h}}{\mathrm{dt}}= & \beta_{h}S_{h}-(\rho E_{h}+\mu_{h})E_{h}\\ \frac{{\mathrm{dI}}_{h}}{\mathrm{dt}}= & \rho_{E_{h}}E_{h}-(\rho I_{h}+\mu_{h}+\alpha )I_{h}\\ \frac{{\mathrm{dR}}_{h}}{\mathrm{dt}}= & \rho_{I_{h}}I_{h}-(\rho R_{h}+\mu_{h})R_{h}\\ \frac{\mathrm{dE}}{\mathrm{dt}}= & n(\rho_{A_{o}}A_{o}+\rho_{I_{v}}I_{v})-(\rho_{e}+\mu_{e})E\\ \frac{\mathrm{dL}}{\mathrm{dt}}= & \rho_{e}E-\left(\rho_{L}+\mu_{L}\left(1+\frac{L}{K}\right)\right)L\\ \frac{\mathrm{dP}}{\mathrm{dt}}= & \rho_{L}L-(\rho_{P}+\mu_{P})P\\ \frac{{\mathrm{dA}}_{h}}{\mathrm{dt}}= & \rho_{p}P+\rho_{A_{o}}A_{o}-(\beta_{v}+D_{v}+\mu A_{h}+℘)A_{h}\\ \frac{{\mathrm{dA}}_{r}}{\mathrm{dt}}= & D_{v}A_{h}-\left(\rho_{A_{r}}+\mu A_{r}+℘\right)A_{r}\\ \frac{{\mathrm{dA}}_{o}}{\mathrm{dt}}= & \rho_{A_{r}}A_{r}-\left(\rho_{A_{o}}+\mu A_{o}+℘\right)A_{o}\\ \frac{{\mathrm{dE}}_{v}}{\mathrm{dt}}= & \beta_{v}A_{h}-\left(\rho_{E_{v}}+\mu E_{v}+℘\right)E_{v}\\ \frac{{\mathrm{dI}}_{v}}{\mathrm{dt}}= & \rho_{E_{v}}E_{v}-\left(\mu I_{v}+℘\right)I_{v} \end{array}$$

### 2.4. Analysis of the Model Outputs

In order to examine the influence of climate variability on the transmission of malaria; we perform the following analysis on the model outputs.

#### 2.4.1. Principal Component Analysis (PCA)

In this study, Principal Component Analysis (PCA) is used to analyse the data generated from the model. The PCA is useful tool for determining similar modes of variability between variables [17–19]. It can also be used to contract umpteen numbers of inter-related variables to a few principal components that accommodate much of the variance in the primary dataset [18]. The analysis helps understand, interpret, and reconstruct large, multivariate datasets, both with spatial extent [20] and at single sites [21]. Here, PCA is applied to identify the climate variables that are coupled with the model outputs. To achieve this, we adopted Statistica software (that is StatSoft Inc., 2013) using the varimax rotation option to obtain a noticeable and clear pattern of loadings.

#### 2.4.2. Wavelet Power Spectrum

Wavelet analysis is a technique used for decomposing a time-series into time-frequency space. The outlook offers valuable perception of the dominant modes of the time-series and how the modes vary with time. Contrary to Fourier analysis, wavelet analysis highlights and identifies the signals whose spectra change with time. In addition, the time-frequency analysis reveals further characteristics such as the periodic components with time progression [3, 7, 22]. The wavelet power spectrum also calculates the distribution of variance between frequency *f* and different time locations *τ*. In order to compare the wavelet power spectrum with simple spectral techniques, the global wavelet spectrum is computed as the time average of the wavelet power spectrum for each frequency component [7]. For a better understanding of this method and analysis, see [23, 24].

Here in this study, we present the basic methods of using wavelet analysis to extricate periodic components from the climate variables and our model outputs. The wavelet analysis investigates the time-scale decomposition of the signal by calculating its spectral attributes as a function of time [22, 25].

#### 2.4.3. Wavelet Cross-Coherence

Time-series analyses have been used to examine the dynamics of several disease epidemics, as it seemed to be the only substitute [26, 27], they are more useful in short-term analyses [28, 29]. They are typically noisy and complex [23]. For these reasons, and in order to qualitatively explore the correspondence of the wavelet spectra of rainfall and temperature on malaria incidence, we examine their cross-coherence spectrum as shown in Figs. (7 and 8) using wavelet cross-coherence analysis.

Wavelet cross-coherence is a technique developed for analysing the coherence and time-phase lag between two time-series as a function of both time and frequency [30]. As defined in Fourier analysis, the univariate wavelet power spectrum can be broaden to analyse statistical relationships between two time-series *x*(*t*) and *y*(*t*) by computing the wavelet coherence, using the formula:

$$R_{x,y}(f,\tau )=\frac{|\langle W_{x,y}(f,\tau )\rangle |}{{|\langle W_{x}(f,\tau )\rangle |}^{1∕2}.{|\langle W_{y}(f,\tau )\rangle |}^{1∕2}},$$

where ⟨ ⟩ denotes smoothing in both time and frequency; *W_x_*(*f, τ*) represents the wavelet transform of series *x*(*t*);*W_y_*(*f, τ*) is the wavelet power transform of series *y*(*t*; and *W_x,y_*(*f, τ*) = *W_x_*(*f, τ*).*W_y_*(*f, τ*) is the cross wavelet power spectrum. The wavelet coherence provides local information about the extent to which two non-stationary signals *x*(*t*) and *y*(*t*) are linearly correlated at a certain period or frequency. The *R_xy_*(*f, τ*) is equal to 1 when there exists a perfect linear relationship at a particular time and frequency between the two signals [23].

## 3. RESULTS AND DISCUSSION

### 3.1. Model Validation

**Fig. (4).**
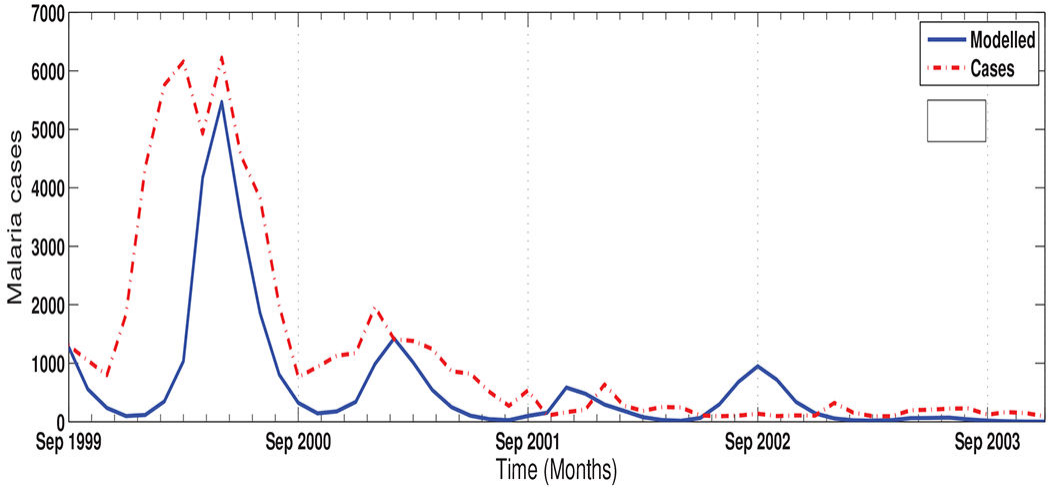
The modelled and reported cases of malaria over KwaZulu-Natal province, South Africa from September 1999 to December 2003.

Comparing the model output with observed data, our results produce a similar curve (*r* = *0.7*) with the observed data as shown in (Fig. 4). For instance both curves highlight the sharp increase in malaria cases in 2000 followed by a decrease in 2001 to 2003. The sudden reduction has been linked to the introduction of dichlorodiphenyltrichloroethane (DDT) in 2000 [31] which was also captured in the model. However, we do notice some discrepancies between the simulated and observed data. The model over estimates malaria incidence as noted in September 2002 by showing almost 1000 infected humans when they were actually less than 200. Also, the malaria prevalence in 2000 picked up around December 1999 and ended in September 2000, while model pick-up time was around January 2000 and ended around October 2000. We consider these as part of the limitations of the model, which might be linked to the lack of other crucial factors affecting malaria in our model.

### 3.2. Malaria and Climate Variability

#### 3.2.1 Correlation Between Climate Variables and Model Outputs

In order to investigate the possible correlation between malaria and climate, we perform principal component analyses on the model outputs.

Our results indicate that there is a process coordinating the relationship between climate variables and malaria. When the process is active, it leads to two principal factors. As indicated in Table 2, the first principal factor (*PF*_1_) shows high loadings on *E_h_* and *I_h_*. Furthermore, the activation of the process increases *E_h_* and *I_h_* but decreases *S_h_*. The second principal factor (*PF*_2_) shows an increase in *S_h_*, decreases the population of *R_h_*. This is logical to the fact that recovered humans can be infected again if bitten by infected mosquito. Our findings here are also consistence with the previous studies of [32] that an increase in infected humans negatively influences the susceptible human population.

#### 3.2.2. Wavelet Time Series Analysis of Climate Variables and Malaria

**Fig. (5).**
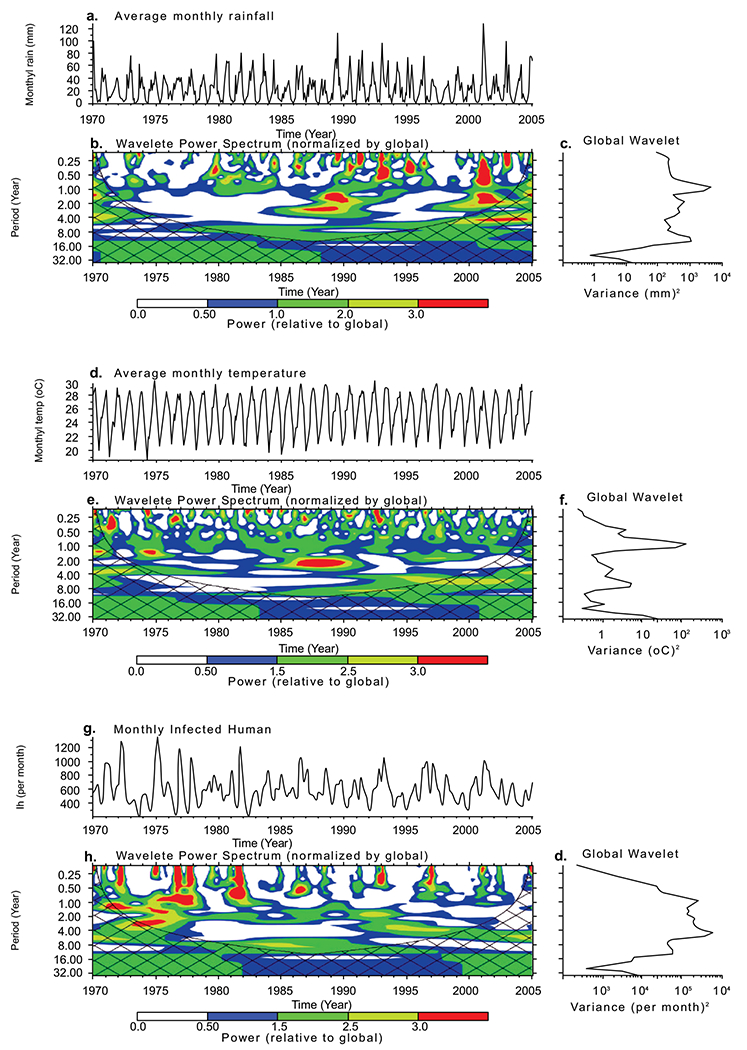
The wavelet analysis of the climate variables of KwaZulu-Natal province from 1970-2005}. The time series of average monthly (a) rainfall, (d) temperature and (g) simulated infected humans. The wavelet power spectrum of (b) rainfall, (e) temperature and (h) Infected humans time series. The cross-hatched region is the cone of influence, where zero padding has reduced the variance and only pattern above the region are considered reliable. The colour code values from blue (low values) to red (high values). The global wavelet power spectrum of (c) rainfall, (f) temperature and (i) Infected humans have been scaled. The black contour line corresponds to 10% significance level, using the global wavelet as the background spectrum.

In general, two dominant peaks are noticed over the province from 1970-2005 as shown in Fig. (5c, f and i). The figures also reveal that 1-year periodicity is highly significant over the study period and describe the largest proportion of the time series. In addition, the monthly time-series, as shown in Fig. (5a, d and g) highlight a recurrent cycle with an apparent 1-year period, and additional components of variability in some years.

The wavelet power spectrum, as illustrated in Fig. (5b, e and h) indicates the decomposition of the series in time (along the x-axis) and period (along the y-axis) scale. The results from the analyses identify a strong annual cycle and ascertained a dominant 1-year periodicity (in red). Additional components of variability at shorter periods are also highlighted in the figures. In particular, the cycles in Fig. (5b) over the study period are noticeable for rainfall between 1986-1996 and 2001-2003, while those of temperature are significantly noticeable from 1986-1991 as revealed in Fig. (5e). However, both climate variables show similar patterns in the cycles from 1986-1991. This is an indication that on a seasonal scale, around this period, both variables increase and decrease simultaneously over the province. Also, the cycle patterns of the infected human are slightly more similar to that of temperature than rainfall. This implies that malaria transmission over the province is more associated with temperature than rainfall.

#### 3.2.3. The Lag and Cross-Correlation of Climate Variability and Malaria

**Fig. (6).**
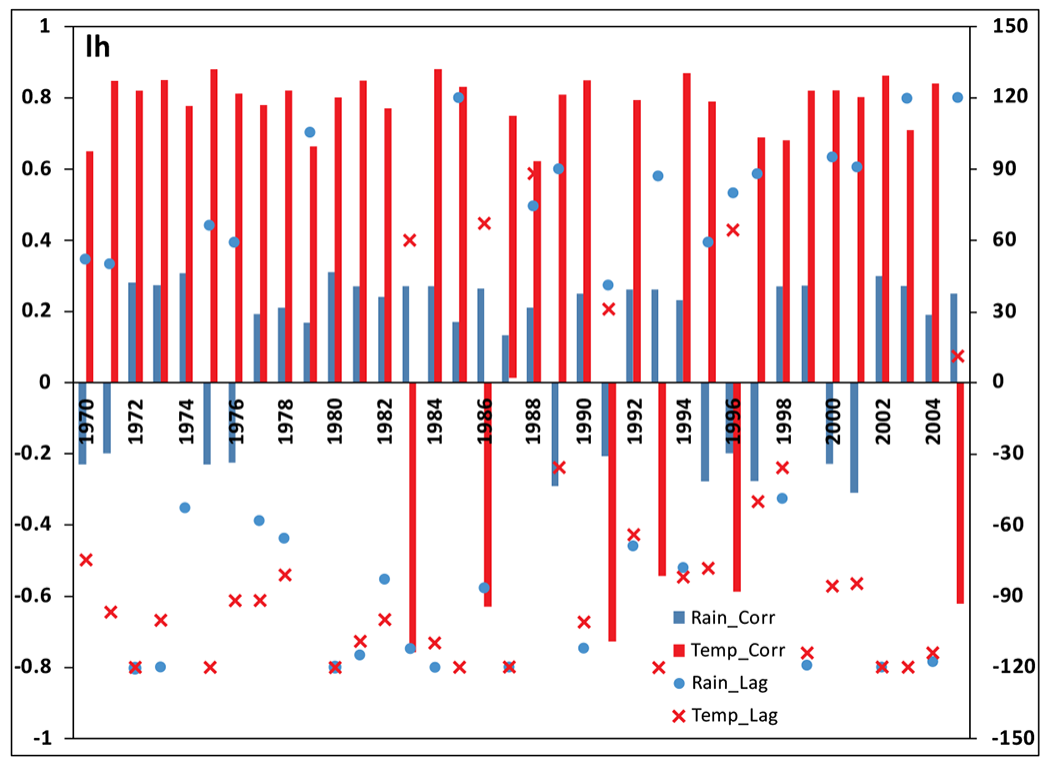
Cross-correlation coefficients of time series of daily climate variables and simulated infected human at several lags.

Also, the results in Fig. (6) show that malaria transmission over KwaZulu-Natal province between 1970 and 2005 is more influenced by temperature than rainfall. For instance, the highest correlation between rainfall and malaria incidence as shown in the figure is below 0.4, while that of temperature is 0.9. In addition, the red and blue bars clearly indicate the existence of both positive and negative correlation between climate variables and infected humans over the study period. However, the positive correlation are more noticeable. This is an indication that both rainfall and temperature contribute positively to the transmission of malaria over the province. Furthermore, an average of 0 to 120-day lag is generally noticed over the years, however, 120-day lag is more associated with temperature than rainfall. Also, in some of the years, rainfall is negatively correlated with number of malaria cases at lags of 0 and 1 month. This is consistence with the previous study of [34], However, our result here contradicts their findings that temperature is weakly correlated at lags of 0 to 4 months. A stronger correlation in the case of temperature is obtained in this study. Other study in line with our findings here is the study of Mohammadkhani *et al* [35]. It is established in their study that the maximum positive cross-correlation was observed between malaria and climatic factors with 1 to 4 months lag [35]. Further studies associated with these findings are [36–38].

**Fig. (7).**
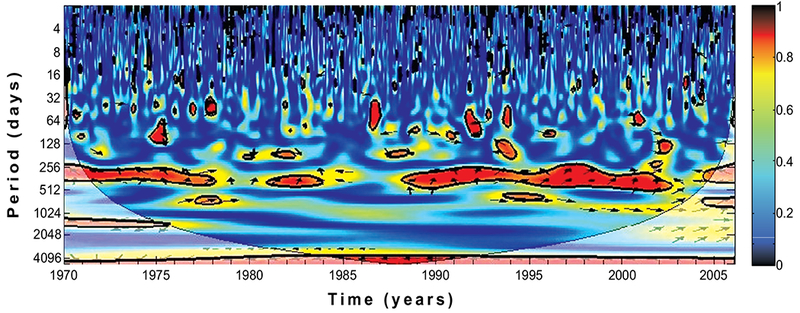
Wavelet coherence of rainfall and simulated infected human over KwaZulu-Natal province from 1970-2005. The arrows indicate the relative phasing of the variables, while the faded regions represent the cone of influence and are not considered for the analyses.

**Fig. (8).**
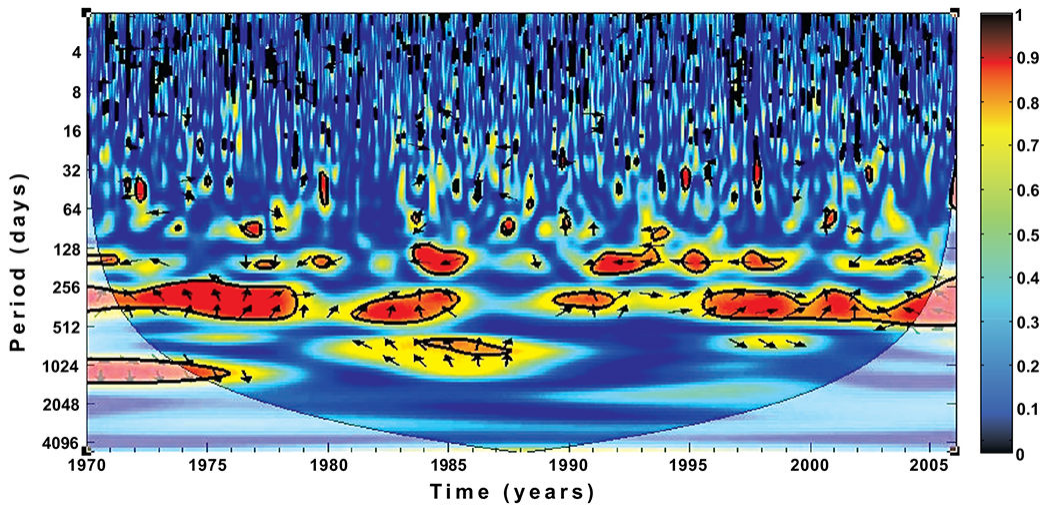
Wavelet coherence of temperature and simulated infected human over KwaZulu-Natal from 1970-2005. The arrows indicate the relative phasing of the variables, while the faded regions represent the cone of influence and are not considered for the analyses.

In addition, the results in Figs. (7 and 8) show a strong relationship and significant cross-coherence between the climate variables and malaria incidence over the province. The results, in line with our previous findings clearly indicate that malaria incidences over the province are more and closely associated with the temperature rather than with rainfall. For instance, the annual cycle is dominated and fairly consistent through the year for both rainfall and temperature. The biennial pattern are additionally noted and more pronounced for temperature than rainfall. Malaria occurrence period is also noticed to fall within 256-512 days on both figures. Focusing more on the two figures, the cross-spectral analysis reveals that the correlation between malaria incidence and rainfall are noticeably stronger between 1971 - 1978 and 1987-2003, while that of temperature are noticeable all through the year. Although we notice a weak in-phase relationship between temperature and malaria incidence on the biennial cycle from 1984-1987 as shown in Fig. (7), no significant cycle or coherence is noted between this period for rainfall (Fig. 8). These results are consistent with the findings of Cazelles *et al* [3] that temperature and rainfall are highly significant on dengue transmission in Thailand. Their findings also made emphasis on the stronger relationship between temperature and dengue fever than that of rainfall. In addition, similar associations were documented for Colombia [39], India [40], and Venezuela [41], among others.

## CONCLUSION

In this study, we have developed a climate-based mosquito-malaria model to examine malaria incidence over KwaZulu-Natal province from 1970 - 2005. The model is developed from the previous study of Abiodun *et al* [13] to investigate the human population dynamics of the province between the study periods.

The model outputs are further analysed with principal component analysis, wavelet power spectrum and wavelet cross-coherence analysis to investigate the relationship between the climate variables and malaria incidence over the province.

Our results ascertain that malaria transmission in the province is seasonal as indicated in previous studies [12, 42–44], The findings also indicate that both temperature and rainfall are responsible for the transmission of the disease. However, malaria is more strongly influenced by temperature than rainfall over the province [42–44], It is further established in our findings that malaria incidence is positively correlated with climate factors between 1 to 4 months lag.

The findings of this study would be useful in early warning or forecasting of malaria transmission over KwaZulu-Natal province. More importantly, attention should be paid to the more expected occurrences of malaria between the periods of 256-512 days.

Currently, the model ignores some other important factors influencing the dynamics of the vector population and malaria transmission over KwaZulu-Natal province. Several studies [2, 45, 46] have highlighted the importance of migration, relative humidity, land cover, irrigation and deforestation mosquito abundance and malaria incidence over a region. We therefore leave these aspects for further studies.

## Figures and Tables

**Table 1.** Parameters of the mosquito-malaria model.

Description	Parameters/Functional Form	Ref.
Number of eggs, *n*(*T_a_*)	−0.061411T^3^_a_ + 38.93T^2^_a_ − 801.27T_a_ + 5391.4	[1]
Egg development rate, *ρ_e_*(*T_w_*)	0.012T^3^_w_ − 0.81T^2^_w_ + 18T_w_ − 135.93	[1]
Larva development rate, *ρ_L_*(*T_w_*)	−0.002T^3^_w_ + 0.14T^2^_w_ − 3T_w_ + 22	[1]
Pupa development rate, *ρ_p_*(*T_w_*)	−0.0018T^3^_w_ + 0.12T^2^_w_ − 2.7T_w_ + 20	[1]
Egg mortality rate, *μ_e_*(*T_w_*)	0.0033T^3^_w_ − 0.23T^2^_w_ − 5.3T_w_ − 40	[1]
Larva mortality rate, *μ_L_*(*T_w_*	0.00081T^3^_w_ − 0.056T^2^_w_ + 1.3T_w_ − 8.6	[1]
Pupa mortality rate, *μ_p_*(*T_w_*	0.0034T^3^_w_ − 0.22T^2^_w_ − 4.9T_W_ − 34	[1]
Gonotrophic rate, *ρA_o_*(*T_a_*)	0.00054T^3^_a_ − 0.038T^2^_a_ − 0.88T_a_	[1]
Mosquito biting rate, Є	0.000203*T_a_*(*T_a_* − 11.7)$\sqrt{42.3-T}$	[37, 40]
Progression rate from *E_v_* to *I_v_*, *μE_v_*(*T_a_*)	$\frac{T_{a}-T_{\min}}{111}$	[37, 43, 50]
Min. temp. for *P. falciparum* survival, *T_min_*	16C	[15, 37, 43]
Proportion of insecticides, *℘*	0.5	Est.
Rate adult mosquito seeks blood meal, *ρA_h_*	0.46	[14, 30]
Rate adult mosquito seeks resting site, *ρA_r_*	0.43	[14, 30]
Probability of human getting infected, *β_1_*	0.533	Nominal
Probability of mosquito getting infected, *β_2_*	0.09	[6, 33, 39]
Natural death rate in human, μ_*h*_	1/49.1/365 per day	[28, 55], Est.
Human recruitment rate, Φ_*h*_	51.67 per day	[28, 55], Est.
Contact rate of mosquito per human, κ	0.6 per day	[13, 39]
Disease induced death rate, *α*	0.05 per day	[33, 39]
Progression rate from *I_v_* to *E_v_*, *ρI_v_*	1/18 per day	[6, 33, 39]
Recovered individuals’ loss of immunity, *ρR_h_*	1/730 per day	[6, 33, 39]

**Table 2.** The principal component analyses (with varimax normalized loadings) showing the possible correlation between the model outputs.

Variable	Principal Factor 1 (PF_1_)	Principal Factor 2 (PF_2_)
*S_h_*	−0.68	0.62
*E_h_*	0.94	0.15
*I_h_*	0.76	−0.42
*R_h_*	0.01	−0.97
Expl. Var	1.92	1.53
Prp. Totl	0.48	0.38
